
JOURNAL INFORMATION
==============================
NLM Title Abbreviation: Wirtschaftsdienst
Journal ID: Wirtschaftsdienst
NLM Journal ID: 101086612

Wirtschaftsdienst (Hamburg, Germany : 1949)

ISSN: 0043-6275

ARTICLE INFORMATION
==============================
PMCID: PMC7368596
PMID: 32834172
DOI: 10.1007/s10273-020-2703-6
Article ID: 2703
Article version: 1
Subjects: Ökonomische Trends

Quo vadis paarinterne Arbeitsteilung post coronam?

The domestic division of labour: Quo vadis post-COVID-19?

Boll Christina boll@dji.de

Schüller Simone schueller@dji.de

grid.424214.5 0000 0001 1302 5619 Abteilung Familie und Familienpolitik, Deutsches Jugendinstitut e.V., Nockherstraße 2, 81541 München, Deutschland
Electronic publication date: 2020 Jul 18
Print publication date: 2020
Volume: 100
Issue: 7
First page: 556
Last page: 558
Copyright: © Der/die Autor(en) 2020
License: Open Access Dieser Artikel wird unter der Creative Commons Namensnennung 4.0 International Lizenz veröffentlicht, welche die Nutzung, Vervielfältigung, Bearbeitung, Verbreitung und Wiedergabe in jeglichem Medium und Format erlaubt, sofern Sie den/die ursprünglichen Autor(en) und die Quelle ordnungsgemäß nennen, einen Link zur Creative Commons Lizenz beifügen und angeben, ob Änderungen vorgenommen wurden.
Die in diesem Artikel enthaltenen Bilder und sonstiges Drittmaterial unterliegen ebenfalls der genannten Creative Commons Lizenz, sofern sich aus der Abbildungslegende nichts anderes ergibt. Sofern das betreffende Material nicht unter der genannten Creative Commons Lizenz steht und die betreffende Handlung nicht nach gesetzlichen Vorschriften erlaubt ist, ist für die oben aufgeführten Weiterverwendungen des Materials die Einwilligung des jeweiligen Rechteinhabers einzuholen.
Weitere Details zur Lizenz entnehmen Sie bitte der Lizenzinformation auf http://creativecommons.org/licenses/by/4.0/deed.de.
License URL: https://creativecommons.org/licenses/by/4.0/

issue-copyright-statement: © The Author(s) 2020

==============================

Das Stichwort „Retraditionalisierung der paarinternen Arbeitsteilung" ist während der COVID-19-Pandemie in aller Munde. Die Diskussion darüber, was nach Corona erwartet werden kann, bewegt sich zwischen zwei Lagern – den Pessimisten, die einen „Backlash" in Form eines massiven Rückfalls in tradierte Verhaltensmuster befürchten, und den Optimisten, die aufgrund der krisenbedingt freigesetzten väterlichen Zeitbudgets sowie der väterlichen Anwesenheit im Homeoffice erwarten, dass Väter mit zuvor relativ geringer Beteiligung die Familienarbeit kennen- und schätzen lernen und auch über die akute Krise hinaus ihren Anteil an der familialen Sorgearbeit dauerhaft erhöhen könnten. Allerdings orientiert sich der derzeitige Diskurs stark an der Zielgröße, ohne die zugrundeliegenden Mechanismen ausreichend in den Blick zu nehmen. Es ist daher mitnichten zu erwarten, dass „künftig dort mehr (väterliche Betreuung) sein wird, wo bisher wenig war und dort eher weniger, wo bisher viel war". Eine Verortung der mittel- bis langfristigen Erwartungen für Deutschland zwischen Chance und „Backlash" bedarf empirisch gestützter Überlegungen dazu, welche Mechanismen der elterlichen Arbeitsteilung durch die Corona-Krise in welcher Weise tangiert werden, und für wie viele (und für welche) Elternpaare von einem unmittelbaren Anpassungsdruck in der Aufteilung der familialen Sorgearbeit während der Kita- und Schulschließungen auszugehen ist.[1]

## Studiendesign und Ergebnisse

Zur Erwartungsbildung der paarinternen Arbeitsteilung nach Corona ziehen wir empirische Evidenz zu paarinternen Ressourcenverhältnissen und Normen vor der Pandemie im Zusammenspiel mit den „Corona-Faktoren" Homeoffice und Systemrelevanz heran. Wir untersuchen zunächst auf Basis des Sozio-oekonomischen Panels (SOEP) 2018[2] die Zusammenhänge zwischen dem väterlichen Kinderbetreuungsanteil im Paar und den in der Literatur einschlägigen Wirkmechanismen Zeitbudgetverhältnis, Einkommensrelation und Geschlechterrolleneinstellungen im Paar (für einen Überblick Boll, 2017). Wir fokussieren uns dabei auf die Kinderbetreuung, denn sie ist (im Vergleich zur Hausarbeit) zeitlich weniger flexibel, zwingend erforderlich und nur bedingt skalierbar, sodass hier im Shut- bzw. Lockdown von Wirtschaft bzw. Schulen und Kitas maximaler Handlungsdruck im Paar besteht, solange das Paar keinen Zugang zur Notbetreuung hat. Theoriegeleitet erwarten wir, dass sich ein Anpassungsdruck auf den väterlichen Betreuungsanteil dann am stärksten entfaltet, wenn sich einseitig sein Zeitbudget für die Familie nennenswert vergrößert, kein Notbetreuungszugang besteht und die Einkommensrelation im Paar (die sich unter der Krise verändern kann) sowie die Geschlechternormen im Paar seiner Verhaltensanpassung nicht entgegenstehen. Zur Illustration werden die Paare in fünf gleich große Gruppen mit aufsteigendem männlichen Betreuungsanteil eingeteilt. Das unterste (oberste) Quintil bilden Elternpaare, in denen der Vater 0 % bis 11 % (43 % bis 100 %) der gesamten Kinderbetreuung übernimmt (vgl. Tabelle 1).

Wie Tabelle 1 zeigt, sind in unserer Untersuchungsstichprobe in der Vor-Corona-Phase die einschlägigen Mechanismen zur Arbeitsteilung im Paar wirksam: Mit steigendem Betreuungsanteil des Vaters sinken der Anteil der Paare mit traditionellem Erwerbsmodell, der väterliche Einkommensanteil sowie der Anteil der Paare mit väterlichem Bildungsvorsprung. Im Gegenzug steigt die Modernität der mütterlichen (ab dem 2. Quintil) und der väterlichen Rollenbilder. Hoffnungen bzw. Befürchtungen zur

1 Der vorliegende Beitrag basiert auf Boll und Schüller (2020).

2 Das SOEP ist eine repräsentative Wiederholungsbefragung von über 25.000 Personen aus rund 16.000 Haushalten (Goebel et al., 2019). Die Untersuchungsstichprobe besteht aus 2.145 heterosexuellen Paaren von 18 bis 65 Jahren mit Kindern unter 13 Jahren, für die jeweils für 2018 Angaben zu allen potenziell für die Arbeitsteilung einflussreichen Merkmalen beider Partner vorliegen. Die Zielgröße, den väterlichen Kinderbetreuungsanteil, fassen wir als prozentualen Anteil an der Summe der Betreuungsstunden im Paar an einem üblichen Werktag. Die Einkommensrelation im Paar messen wir über den väterlichen Einkommensanteil an der Summe der monatlichen Bruttoerwerbseinkommen der Partner. Das relative Zeitbudget im Paar erfassen wir über die Erwerbskonstellation im Paar (diverse Kategorien). Die Modernität der Geschlechterrollen definieren wir als Zustimmung zu dem Statement „Am besten ist es, wenn der Mann und die Frau beide gleichviel erwerbstätig sind und sich beide in gleichem Maße um Haushalt und Familie kümmern".

Tabelle 1
**Paareigenschaften nach Quintilen der väterlichen Betreuungsbeteiligung im Paar**

| Quintile der väterlichen Betreuungsbeteiligung | Traditionelles Erwerbsmodell in % | Anteil Vater am gesamten Erwerbseinkommen des Paares, Mittelwert, in % | Vater mit höherem Bildungsabschluss als Mutter, in % | Modernität der Rollenbilder auf einer Skala von 0 bis 1, Mittelwert | |
|---|---|---|---|---|---|
| | | | | Mutter | Vater |
| Q1 (0 % bis 11 %) | 81,9 | 80,2 | 40,0 | 0,71 | 0,64 |
| Q2 (12 % bis 20 %) | 88,0 | 81,7 | 35,1 | 0,64 | 0,65 |
| Q3 (21 % bis 28 %) | 79,9 | 73,3 | 35,3 | 0,71 | 0,74 |
| Q4 (29 % bis 42 %) | 68,5 | 65,1 | 34,1 | 0,75 | 0,75 |
| Q5 (43 % bis 100 %) | 46,1 | 52,9 | 29,4 | 0,79 | 0,79 |
| Insgesamt | 73,6 | 71,2 | 34,9 | 0,72 | 0,71 |

Anmerkungen: N = 2.145. Als traditionelle Erwerbsmodelle gelten das männliche Allein- und Hauptverdienermodell.

Quelle: eigene Berechnungen auf Basis des SOEP 2018 (soep.v35), gewichtet.

Änderung der unbezahlten Arbeit im Paar, die sich allein an der Zielgröße väterlicher Betreuungsbeteiligung selbst ausrichten, könnten daher enttäuscht werden, wenn die Wirkmechanismen Zeitbudgetverhältnis, Einkommensrelation und Geschlechterrolleneinstellungen im Paar (und die pandemiebedingten Änderungen derselben) nicht systematisch mitgedacht werden.

Wir gehen davon aus, dass während der Pandemie zwei berufliche Charakteristika an Relevanz für die Arbeitsteilung im Elternpaar gewinnen – erstens die Möglichkeit der Eltern, im Homeoffice (HO) zu arbeiten, und zweitens die Systemrelevanz (SR) ihrer ausgeübten Berufe.[3] Wir unterscheiden zwischen nicht-systemrelevanten Berufen (NSR), systemrelevanten Berufen im Gesundheits- und Pflegebereich (SRGes) und systemrelevanten Berufen im Infrastrukturbereich (SRInf). Letztgenannte Differenzierung bestimmt den elterlichen Zugang zur Notbetreuung in Kitas und Schulen. Auf Basis der institutionellen Regelungen in den Bundesländern nehmen wir an, dass Eltern Zugang zu Notbetreuung haben, wenn beide Eltern in einem SRInf-Beruf sind oder mindestens ein Elternteil in einem SRGes-Beruf beschäftigt ist. Gemäß dieser Restriktion haben in unserer Stichprobe 13,1 % der Paare Zugang zu Kita-Notbetreuung. Wir nehmen an, dass dieses Szenario für den Zeitraum Mitte März bis Ende April 2020 gegolten hat.

Bei 10,9 % der Paare ist nur die Mutter, bei weiteren 13,2 % der Paare nur der Vater in einem SRInf-Beruf tätig; der/die Partner*in ist hier jeweils in einem NSR-Beruf beschäftigt. Bei der Mehrheit der Paare (62,77 %) sind beide Partner in nicht-systemrelevanten Berufen beschäftigt. Bei 22,8 % unter allen betrachteten Paaren haben beide Elternteile Zugang zum Homeoffice, bei 24,5 % ist dies nur für den Vater und in 13,2 % nur für die Mutter der Fall. Bei 39,6 % der Paare hat kein Elternteil Zugang zum Homeoffice.

In einem zweiten Schritt validieren wir die Relevanz der berufsbezogenen Corona-Faktoren Systemrelevanz und Homeoffice-Potenzial für die Arbeitsteilung auf Basis ihres theoretischen Einflusses auf die genannten Wirkmechanismen. Homeoffice kann durch eingesparte Pendelzeiten und mehr Flexibilität das Zeitbudget von Vätern tendenziell vergrößern. In NSR-Berufen kann die HO-Fähigkeit jobsichernd wirken. In systemrelevanten Berufen (vor allem in SRGes-Berufen) kann eher nicht im Homeoffice gearbeitet werden, in dem Fall sind Väter zuhause nicht präsent. Zudem besteht in SR-Berufen eher kein Jobrisiko.

Auf Basis unserer Analysen quantifizieren wir insgesamt 6,7 % der Paare als potenzielle Hoffnungsträger, von denen wir einen Anstieg des väterlichen Kinderbetreuungsanteils im Paar am ehesten erwarten. Dies sind Paare in drei Konstellationen: Solche, in denen nur die Mutter in einem SRInf-Beruf arbeitet und nur der Vater HO-fähig ist (= 1,0 % aller Paare, vgl. Abbildung 1), solche, in denen nur der Vater in einem SRInf-Beruf tätig ist und beide Partner HO-fähig sind (= 2,8 % aller Paare, vgl. Abbildung 1) und Paare, in denen kein Partner in einem SR-Beruf arbeitet, aber beide HO-fähig und in Vollzeitjobs beschäftigt sind (2,92 % aller Elternpaare). Letztere sind eine kleine Teilgruppe der 14,6 % Elternpaare, in denen kein Elternteil einen systemrelevanten Beruf ausübt und beide Eltern einen Zugang zum Homeoffice haben (vgl. Abbildung 1). Eltern mit Zugang zur Notbetreuung werden dabei nicht als Hoffnungsträger betrachtet, da der Anpassungsdruck im Paar hier (zumindest teilweise) durch das Angebot externer Betreuung entweichen kann. Ebenso sehen wir davon ab, Paare als Hoffnungsträger zu bezeichnen, in denen

3 Wir verwenden das in Alipour, Falck und Schüller (2020) auf Basis der BIBB/BAuA-Erwerbstätigenbefragung 2018 berechnete Maß des beruflichen Homeoffice-Potenzials. Zur Einordnung der Systemrelevanz von Berufen orientieren wir uns an der Klassifikation von Koebe et al. (2020) auf Basis der KldB 2010 (3-Steller).

Abbildung 1

**Homeoffice-Potenzial und Systemrelevanz von Elternpaaren**

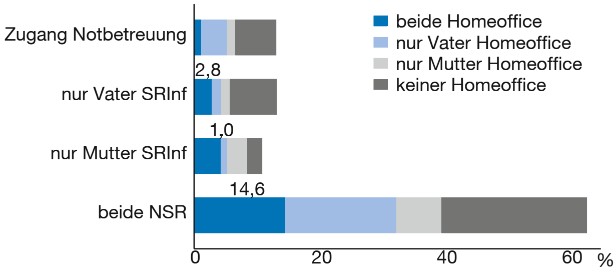

Anmerkungen: N = 2.145. NSR = nicht-systemrelevante Berufe; SRInf = systemrelevante Berufe im Infrastrukturbereich.

Quelle: eigene Berechnungen auf Basis des SOEP 2018 (soep.v35), gewichtet.

ein Elternteil aufgrund nicht-systemrelevanter Beschäftigung ohne Zugang zum Homeoffice ein erhöhtes Risiko krisenbedingter Kurzarbeit oder Arbeitslosigkeit erfährt, sodass es hier zu erheblichen finanziellen Verwerfungen für die Familie kommen kann.[4] Die empirische Evidenz zu vergangenen Krisen gibt zudem nur begrenzt (vor allem bei Vätern) Anlass zur Erwartung, dass die durch Arbeitslosigkeit gewonnenen Zeitbudgets überwiegend in die unbezahlte Familienarbeit fließen.

## Fazit: Die Lage ist ernst, aber nicht hoffnungslos

Der geringe Anteil der abgeschätzten Hoffnungsträger unter den Elternpaaren bietet keinen Anlass zu Euphorie. Die Lage ist durchaus ernst, aber sie ist nicht hoffnungslos. Wir erwarten eine umso größere Nachhaltigkeit der Verhaltensänderungen, je länger sich die Rückkehr zum Regelbetrieb in Kitas und Schulen hinzieht: Je länger neue Routinen eingeübt werden können, desto stärker schmilzt ein etwaiger Produktivitätsvorteil der Mutter bei der Kinderbetreuung ab und desto nachhaltiger sollte die Verhaltensänderung dementsprechend sein. Die Nachhaltigkeit der Verhaltensänderungen wird durch die breitenwirksamen Effekte von Corona zugleich gestärkt und begrenzt. Die Pandemie trifft die Familien in ihrer ganzen Breite. Dies spricht für die Nachhaltigkeit der Veränderungen, da es für einen Wandel dominanter sozialer Normen auf der Metaebene einer kritischen Masse von Unterstützenden bedarf. Allerdings ist die Wahrscheinlichkeit, dass in Familien mit älteren Kindern bereits tradierte Verhaltensmuster aufgebrochen und geändert werden, geringer als bei Familien kurz nach der Geburt eines (vor allem des ersten) Kindes; unter anderem deshalb lassen sich väterliche Verhaltenseffekte aus der Forschung zu den Vatermonaten im Elterngeld nur bedingt auf die Corona-Situation übertragen. Wir erwarten, dass die Nachhaltigkeit der Verhaltensänderungen im Paar durch Multiplikatoreffekte väterlicher Führungskräfte in den Betrieben begünstigt wird. Väter könnten als „positive role models" ihre Erfahrungen mit und Neubewertungen von unbezahlter Sorgearbeit an ihre eigenen Mitarbeitenden und Kolleg*innen weitergeben und somit einen kulturellen Wandel in den Unternehmen vorantreiben. Quantitativ ist dieser Effekt nicht unerheblich: 9 % aller vollzeitbeschäftigten Väter in unserer Stichprobe sind Führungskräfte; von denjenigen unter ihnen mit Zugang zum Homeoffice sind es sogar 11,4 %.

Grundsätzlich steht zu vermuten, dass sich Lerneffekte nicht nur in der privaten Sphäre, sondern vor allem auch im betrieblichen Bereich vollziehen. Diese sollten Feedbackeffekte in die Familien erzeugen und so den angestoßenen Wandel mittelfristig weiter stützen. Wir gehen davon aus, dass der durch Corona ausgelöste Schub mobilen Arbeitens die Geschlechtergleichstellung im Paar weiter voranbringt. Durch Corona sind die Kompetenzen und das Erfahrungswissen der Bevölkerung im Umgang mit digitalen Tools sprunghaft gestiegen. Dieser Wissenssprung bezüglich des Zugangs sowie der Lern- und Arbeitsproduktivität im Homeoffice ist unumkehrbar und wird die Präsenzkulturen in den Unternehmen weiter zurückdrängen. Gelingt dies, könnten davon insbesondere mütterliche Karrieren, aber auch Karrieren junger familienaktiver Väter profitieren.

4 Ändern wir unsere Annahmen zum Zugang der Eltern zu Notbetreuung, d.h. nehmen wir die Zwei-Eltern-Regel auch in SRGes-Berufen an, kommen als zusätzliche Hoffnungsträger nur Paare infrage, bei denen die Mutter den SRGes-Beruf ausübt, der Vater in einem HO-fähigen NSR-Beruf beschäftigt ist und beide Vollzeit arbeiten; dies sind 0,9 % aller Paare. Damit steigt die Zahl der Hoffnungsträger auf insgesamt 7,6 % der Paare.

Dr. Christina Boll leitet die Abteilung Familie und Familienpolitik am Deutschen Jugendinstitut in München.

Dr. Simone Schüller ist dort wissenschaftliche Referentin.


Literatur

Alipour, J.-V., O. Falck und S. Schüller (2020), Germany’s Capacities to Work from Home, CESifo Working Paper, 8227.
Boll, C. (2017), Die Arbeitsteilung im Paar — Theorien, Wirkungszusammenhänge, Einflussfaktoren und exemplarische empirische Evidenz, Expertise im Rahmen des Zweiten Gleichstellungsberichts der Bundesregierung, Hamburg, 30.3.2016, http://www.gleichstellungsbericht.de/de/article/51.expertisen.html (30. Juni 2020).
Boll, C., und S. Schüller (2020), Die Lage ist ernst, aber nicht hoffnungslos — empirisch gestützte Überlegungen zur elterlichen Aufteilung der Kinderbetreuung vor, während und nach dem COVID-19 Lockdown, SOEPpapers on Multidisciplinary Panel Data Research, 1089, https://www.diw.de/documents/publikationen/73/diw_01.c.792058.de/diw_sp1089.pdf (30. Juni 2020).
Goebel J Grabka M M Liebig S Kroh M Richter D Schröder C Schupp J The German Socio-Economic Panel Study (SOEP), Jahrbücher für Nationalökonomie und Statistik Journal of Economics and Statistics 2019 239 2 345 360
Koebe J Samtleben C Schrenker A Zucco A Systemrelevant und dennoch kaum anerkannt: Das Lohn- und Prestigeniveau unverzichtbarer Berufe in Zeiten von Corona DIW aktuell 2020 28 24
